# Signaling Pathway of Histamine H_1_ Receptor-Mediated Histamine H_1_ Receptor Gene Upregulation Induced by Histamine in U-373 MG Cells

**DOI:** 10.3390/cimb43030088

**Published:** 2021-09-24

**Authors:** Hiroyuki Mizuguchi, Yuko Miyamoto, Takuma Terao, Haruka Yoshida, Wakana Kuroda, Yoshiaki Kitamura, Noriaki Takeda, Hiroyuki Fukui

**Affiliations:** 1 Laboratory of Pharmacology, Faculty of Pharmacy, Osaka Ohtani University, Osaka 584-8540, Japan; hfukui@tokushima-u.ac.jp; 2 Department of Molecular Pharmacology, Institute of Biomedical Sciences, Tokushima University Graduate School, Tokushima 770-8505, Japan; c400632032@stud.tokushima-u.ac.jp (Y.M.); c400603054@stud.tokushima-u.ac.jp (T.T.); c400502053@stud.tokushima-u.ac.jp (H.Y.); 3 Department of Otolaryngology, Institute of Biomedical Sciences, Tokushima University Graduate School, Tokushima 770-8505, Japan; c400932010@stud.tokushima-u.ac.jp (W.K.); ykitamura@tokushima-u.ac.jp (Y.K.); takeda@tokushima-u.ac.jp (N.T.); 4 Medical Corporation Kinshukai, Osaka 558-0011, Japan

**Keywords:** gene upregulation, histamine, histamine H_1_ receptor, splice valiant, transcription regulation

## Abstract

Histamine H_1_ receptor (H1R) is one of the targets of histamine in the nervous system and the peripheral tissues. Protein kinase Cδ (PKCδ) signaling is involved in histamine-induced upregulation of H1R gene expression in HeLa cells. Histamine also upregulates H1R gene expression in U-373 MG cells. However, the molecular signaling of this upregulation is still unclear. Here, we investigated the molecular mechanism of histamine-induced H1R gene upregulation in U-373 MG cells. Histamine-induced H1R gene upregulation was inhibited by H1R antagonist *d*-chlorpheniramine, but not by ranitidine, ciproxifan, or JNJ77777120, and H2R, H3R, or H4R antagonists, respectively. Ro-31-8220 and Go6976 also suppressed this upregulation, however, the PKCδ selective inhibitor rottlerin and the PKCβ selective inhibitor Ly333531 did not. Time-course studies showed distinct kinetics of H1R gene upregulation in U-373 MG cells from that in HeLa cells. A promoter assay revealed that the promoter region responsible for H1R gene upregulation in U-373 MG cells was different from that of HeLa cells. These data suggest that the H1R-activated H1R gene expression signaling pathway in U-373 MG cells is different from that in HeLa cells, possibly by using different promoters. The involvement of PKCα also suggests that compounds that target PKCδ could work as peripheral type H1R-selective inhibitors without a sedative effect.

## 1. Introduction

Histamine is involved in physiological and pathological functions in both peripheral tissue and the nervous system, and its actions are mediated by four histamine receptor subtypes, H_1_, H_2_, H_3_, and H_4_ receptors [1,2]. Histamine acts as a neurotransmitter in the nervous system. On the other hand, histamine acts as a chemical mediator in type I allergic reactions and gastric acid secretion in peripheral tissues. H1R is expressed in airway epithelial, endothelial, and involved in allergy-including allergic rhinitis, atopic dermatitis, anaphylaxis, and asthma [3]. It was reported that H1R mRNA expression increased in epithelial, endothelial, and neural cells of the nasal mucosa in patients with occupational rhinitis [4,5]. Upregulation of H1R gene expression was also observed in patients with allergic rhinitis, and H1R binding in the nasal mucosa increased during the development of nasal allergies [6,7,8]. We have shown that prophylactic administration of antihistamines decreased nasal mucosal H1R gene upregulation with alleviation of nasal symptoms in patients with pollinosis [9]. In addition, by using environmental exposure units, we also showed that pre-administration of ebastine, an antihistamine, downregulated H1R gene expression before pollen exposure and then inhibited pollen-induced nasal symptoms and pollen-induced upregulation of H1R gene expression in the nasal mucosa of patients with pollinosis [10]. These findings suggest that the H1R gene is an allergy-sensitive gene, i.e., its expression level affects the severity of symptoms, and compounds that target the H1R gene expression pathway might be useful for developing new effective anti-allergic medications. Consistent with this hypothesis, we have shown that compounds that suppressed the upregulation of H1R gene expression alleviated nasal symptoms in allergy model rats [11,12,13,14]. We showed that the stimulation of H1R with histamine causes upregulation of H1R in HeLa cells [15], and the protein kinase Cδ (PKCδ)/heat shock protein 90 (Hsp90) pathway was involved in this H1R-mediated H1R gene expression [16,17].

In the nervous system, H1R is expressed in neuronal and glial cells, and it is involved in the thermal regulation, memory and learning, sleep–wake cycle, and food intake. Histamine-induced H1R upregulation was reported in primary cultured astrocytes [18]. On the contrary, histamine-induced H1R internalization in U-373 MG astrocytoma cells was reported [19]. We showed that stimulation with phorbol-12-myristate-13-acetate (PMA) upregulated H1R gene expression in U-373 MG cells, suggesting that the H1R gene expression signaling pathway is PKC-dependent [20]. However, little is known about the molecular signaling pathway of histamine-induced upregulation of H1R gene expression in U-373 MG cells. 

In the present study, we sought to investigate the signaling pathway of histamine-induced H1R gene upregulation in U-373 MG cells. Our data revealed that histamine-induced upregulation of H1R gene expression in U-373 MG cells is mediated by H1R. We also showed that PKCα was involved in the H1R signaling pathway in U-373 MG cells, whereas PKCδ is involved in H1R gene upregulation in HeLa cells. Stimulation with histamine caused a rapid and transient increase in the H1R mRNA level in U-373 MG cells, which is different from that in HeLa cells, suggesting that H1R gene transcription was regulated by the different promoter from that in HeLa cells.

## 2. Materials and Methods

### 2.1. Cell Culture

HeLa cells were cultured at 37 °C under a humidified 5% CO_2_/95% air atmosphere in MEM-alpha medium (GIBCO, Carlsbad, CA, USA) containing 8% fetal calf serum and 1% antibiotic-antimycotic (Nacalai Tesque, Kyoto, Japan). U-373 MG (Uppsala) cells were cultured in DMEM (GIBCO) with 10% FBS and 1% antibiotic-antimycotic. The cells were cultured to 70% confluence in 6-well dishes. The serum was removed for 24 h at 37 °C before treatment with histamine or PMA (Sigma, St. Louis, MO, USA). At given time intervals after stimulation with 1 to 100 μM histamine or 100 nM PMA, the cells were harvested with 700 μL of RNAiso Plus (Takara Bio Inc., Kyoto, Japan), mixed with 210 μL of chloroform, and centrifuged at 15,000 rpm for 15 min at 4 °C. For the dose-dependent studies, the U-373 MG cells were treated with or without histamine (1–100 μM) for 3 h. For the inhibition studies, the U-373 MG cells were treated with or without isoform-selective histamine receptor antagonists (1 μM each) or isozyme-selective PKC inhibitors (1 and 10 μM) 1 h before histamine stimulation.

The aqueous phase containing total RNA was collected, and RNA was precipitated by the addition of isopropyl alcohol. After centrifugation at 15,000 rpm for 15 min at 4 °C, the resulting RNA pellet was washed with ice-cold 70% ethanol. Total RNA was resolved in 10 μL of diethylpyrocarbonate-treated water (DEPC-DW), and 5 μg of each RNA sample was used for the reverse transcription reaction.

### 2.2. Animal Experiments

Six-week-old male Brown Norway rats (200–250 g; Japan SLC, Hamamatsu, Japan) were used in this study. The rats were allowed free access to water and food and were kept in a room at 25 ± 2 °C and 55 ± 10% humidity with a 12 h light/dark cycle. They were divided into two groups of four rats each: a control group and a group sensitized with TDI (Wako Pure Chemical Industries Ltd., Tokyo, Japan). The rats were sensitized with toluene-2, 4-diisocyanate (TDI) by the method described by Kitamura et al. [21], with slight modifications. Briefly, 10 μL of a 10% (*v*/*v*) solution of TDI in ethyl acetate (Wako Pure Chemical, Osaka, Japan) was applied bilaterally to the nasal vestibule once a day for 5 consecutive days. This sensitization procedure was then repeated after a 2-day interval. Nine days after the second sensitization, 10 μL of 10% TDI solution was again applied to the nasal vestibule in order to provoke nasal allergic-like symptoms. The control rats were treated with ethyl acetate only according to the same schedule. All procedures involving animals were conducted following the Guidelines for Animal Experiments approved by the Ethical Committee for Animal Studies of the School of Medicine of the University of Tokushima (the projection identification number, 08111; date of approval, 12 August 2008).

### 2.3. Real-Time Quantitative RT-PCR

In animal experiments, at given time intervals after provocation, rats were sacrificed, and nasal mucosa and trigeminal ganglion were removed, collected in RNAlater (Applied Biosystems, Foster City, CA, USA), and stored at −80 °C until used. Tissue was homogenized in 10 volumes (*w*/*v*) of RNAiso Plus by using a Polytron (Model PT-K; Kinematica AG, Littau/Luzern, Switzerland). Homogenates were then mixed with 0.3 (*v*/*v*) volumes of chloroform and centrifuged at 15,000 rpm for 15 min at 4 °C. Aqueous phases containing RNA were then transferred to fresh tubes, and RNA was precipitated by adding isopropanol and centrifuged at 15,000 rpm for 15 min at 4 °C. The RNA samples were then dissolved in DEPC-DW, and the RNA samples (8 μg) were reverse-transcribed to cDNA by using a PrimeScript RT Reagent Kit (Takara). TaqMan primers and probe were designed using the Primer Express software (Applied Biosystems). Real-time PCR was conducted using a GeneAmp 7300 sequence detection system (Applied Biosystems). The sequences of the primers and TaqMan probe are listed in Table 1. In order to standardize the starting material, endogenous control human GAPDH control reagent (Applied Biosystems) was used, and data were expressed as a ratio of GAPDH mRNA.

### 2.4. Determination of Luciferase mRNA Using Promoter Assay System

In order to assess the rapid change in transcriptional activity, we investigated the kinetics of luciferase mRNA expression in response to histamine/PMA stimulation in both HeLa cells and U-373 MG cells using the following promoter assay system. The construction of human H1R reporter plasmid containing the promoter region of H/I/K splice variant was described previously [15]. Human H1R reporter plasmids containing the putative promoter region of A/K, B/K, or F/K splice variants were constructed as follows: 2.5 kb DNA fragments 5’-upstream of exon A, B, and F [22] were PCR amplified by using primers shown in Table 2. The fragments were subcloned into the NheI-HindIII site of the promoterless *Photinus pyralis* luciferase (P_luc_) reporter plasmid pGL3-Basic vector (Promega, Madison, WI, USA). HeLa cells or U-373 MG cells cultured in 6-well culture plates were then cotransfected with the reporter plasmid containing the promoter region of each splice variant and the internal control plasmid pRL-MPK in a ratio of 100:1 for U-373 MG cells and pRL-TK in a ratio of 20:1 for HeLa cells using PolyFect transfection reagent (Qiagen) according to the manufacturer’s instructions. The cells were stimulated with histamine or PMA for the indicated time in the same medium, and total RNA was isolated. After removal of residual plasmid DNA by DNase, P_luc_ mRNA was determined by real-time quantitative RT-PCR as described above using the primers and probes shown in Table 1. In order to standardize the starting material, *Renilla reniformis* luciferase (R_luc_) mRNA was determined, and data were expressed as a ratio of R_luc_ mRNA.

### 2.5. RT-PCR

After starvation in serum-free medium for 24 h, the cells were harvested, and total RNA was isolated, as described above. Total RNA (2 μg) was reverse-transcribed to cDNA in the reaction mixture of 20 μL. Three μL of aliquot was used for the endpoint-PCR analysis. The PCR conditions were 35 cycles of 94 °C for 30 s, 58 °C for 30 s, and 72 °C × 1 min followed by a final extension at 72 °C for 10 min. The nucleotide sequences of the primers used are as follows: the forward primer for H/I/K splice variant 5′-TTAAGAAGCCCATCATGGAGAA-3′ and the reverse primer, 5′-TTATCTTCCATCT-AGTGTAACTTGTTCA-3′; the forward primer for B/K splice variant 5′-CAAACTT-TCCCCGGAGCCG-3′ and reverse primer, 5′-TGTTGCCCTCACACATCTTGTCTTC-3′.

### 2.6. Statistical Analysis

The results are shown as mean ± SEM. Statistical analysis was performed by ANOVA with Tukey–Kramer’s test using GraphPad Prism software (GraphPad Software Inc., La Jolla, CA, USA). Values of *p* < 0.05 are considered to be statistically significant.

## 3. Results and Discussion

### 3.1. Histamine-Induced Upregulation of H1R Gene Expression Is Mediated by H1R Activation, and PKCα Is Involved in Its Signaling Pathway in U-373 MG Cells

**Figure 1:**
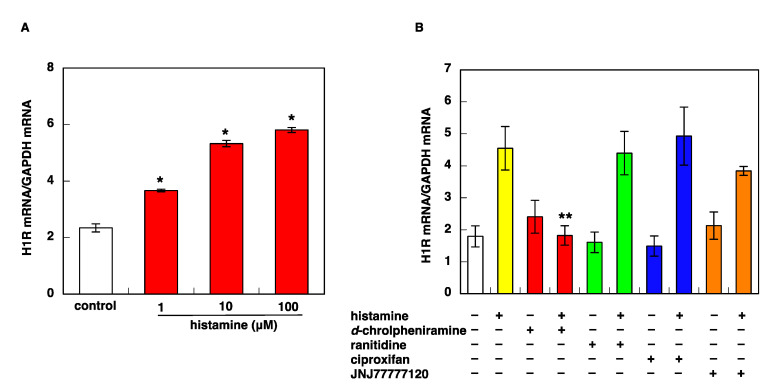
Stimulation with histamine causes upregulation of H1R mRNA expression through H1R activation in U-373 MG cells. (**A**) Dose-dependent study of histamine-induced H1R mRNA upregulation. U-373 MG cells were starved in serum-free medium for 24 h before treatment with various concentrations of histamine. H1R mRNA was determined by quantitative RT-PCR 3 h after histamine treatment. Data are represented as means ± SEM (*n* = 3). * *p* < 0.05 vs. control. (**B**) Effects of histamine receptor antagonists on histamine-induced H1R mRNA upregulation. Histamine receptor antagonists (1 μM each) were treated 1 h before stimulation with 100 μM histamine. Antagonists used are as follows, *d*-chlorpheniramine (H1R antagonist), ranitidine (H2R antagonist), ciproxifan (H3R antagonist), and JNJ777120 (H4R antagonist). Data are represented as means ± SEM (*n* = 9). ** *p* < 0.01 vs. histamine. For statistical analysis, one-way ANOVA with Tukey–Kramer’s test was used.

We previously demonstrated that histamine upregulated H1R gene expression through the activation of H1R, and the PKCδ/Hsp90/ERK signaling pathway is involved in H1R mRNA upregulation [16,17]. The stimulation of U-373 MG cells with histamine also upregulated H1R gene expression in a dose-dependent manner (Figure 1A). This upregulation of H1R gene expression was inhibited by pretreatment with H1R antagonist *d*-chlorpheniramine, but not by ranitidine, ciproxifan, or JNJ77777120, an H2R, H3R, or H4R antagonist, respectively. (Figure 1B). These data indicate that histamine-induced upregulation of H1R gene expression in U-373 MG cells is mediated by H1R, which is the same as in the case of HeLa cells [15]. 

Histamine-induced upregulation of H1R gene expression was suppressed by pan-PKC inhibitor Ro-31-8220. It was reported that H1R is in an equilibrium state between an active form and an inactive form, and a constant level of signal always operates even without histamine stimulation [23,24]. Data showed that 10 μM of Ro-031-8220 suppressed H1R gene upregulation below the control level, suggesting that it inhibits the constitutive activity of H1R, and PKC signaling is the primary signal pathway for H1R-activated H1R gene expression in U-373 MG cells. PKCα/βI inhibitor Go6976 also suppressed histamine-induced H1R gene upregulation. As the suppressive activity of 10 μM of Go6976 is the same as that when 1 μM Go6976 was used, it is likely that 1 μM of Go6976 is enough to suppress H1R gene expression. Ly333531 (10 μM), a PKCβ inhibitor, showed no inhibition for histamine-induced H1R gene upregulation. It was also reported that PKCβ does not express in U-373 MG cells [25]. Therefore, the effect of Go6976 is achieved through PKCα inhibition. No further suppression of H1R gene expression by the treatment with 10 μM of Go6976 suggests the involvement of the additional PKC isozyme(s) in addition to PKCα in U-373 MG cells.

**Figure 2:**
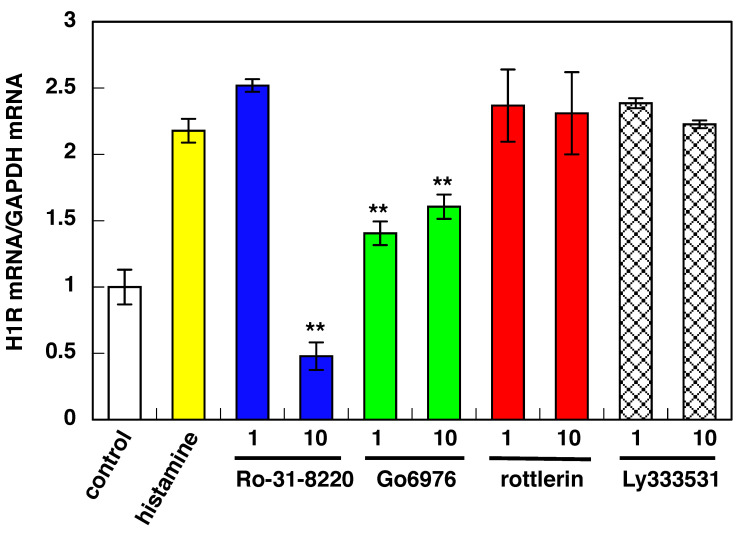
Effect of PKC isozyme-selective inhibitors on histamine-induced upregulation of H1R gene expression in U-373 MG cells. Isozyme-selective PKC inhibitors were treated 1 h before stimulation with 100 μM histamine. After stimulation, total RNA was isolated, and the H1R mRNA level was determined by real-time RT-PCR. Inhibitors used are as follows: Ro-31-8220 (pan-PKC inhibitor), Go6976 (PKCα/β1 inhibitor), rottlerin (PKCδ inhibitor), and Ly333531 (PKCβ inhibitor). Data are represented as means ± SEM (*n* = 9). ** *p* < 0.01 vs. histamine. For statistical analysis, one-way ANOVA with Tukey–Kramer’s test was used.

To date, 12 PKC isoforms have been identified, and they are divided into three subgroups: conventional PKC (α, βI, βII, and γ), novel PKC (δ, ε, η, and θ), and atypical PKC (z and ι/λ) based on their structures and cofactor requirements [26]. PKCμ and PKCν were recently identified isozymes [27]. It was reported that PKC α, γ, δ, ε, z, ι/λ, and m isozymes were expressed in U-373 MG cells [25]. Our data suggest that PKCδ and PKCβ are not involved in histamine-induced H1R gene expression in U-373 MG cells (Figure 2). Conclusively, PKCα is mainly involved in the histamine-induced H1R gene expression pathway in U-373 MG cells. In addition to PKCα, the additional PKC isozyme(s) except PKCδ or PKCβ could be involved in H1R-activated H1R gene expression in U-373 MG cells. Identification of the additional PKC isozyme(s) is under investigation in our laboratory.

### 3.2. Stimulation with Histamine Caused a Rapid and Transient Increase in H1R mRNA Levels in U-373 MG Cells

**Figure 3:**
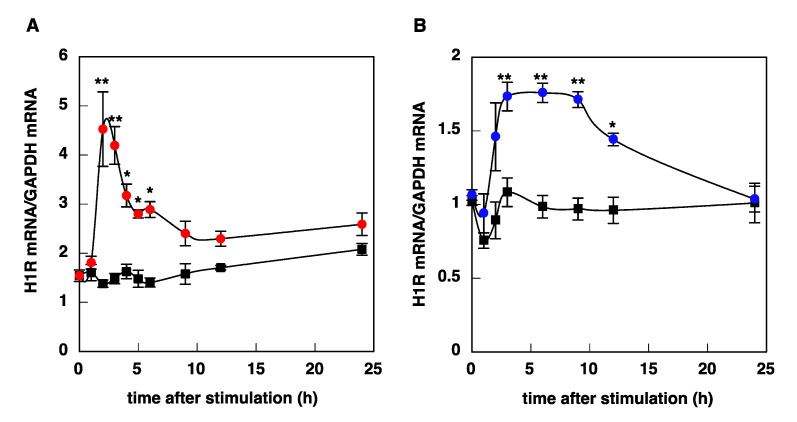
Time course of histamine-induced upregulation of H1R mRNA in U-373 MG cells (**A**) and HeLa cells (**B**). At given time intervals after stimulation with 100 μM (U-373 MG cells) or 10 μM (HeLa cells) histamine, total RNA was isolated, and H1R mRNA was determined by real-time quantitative RT-PCR. Closed circles, histamine; closed squares, control. Data are expressed as means ± SEM (*n* = 8–12). * *p* < 0.05 vs. control; ** *p* < 0.01 vs. control. For statistical analysis, two-way ANOVA with Tukey–Kramer’s test was used.

**Figure 4:**
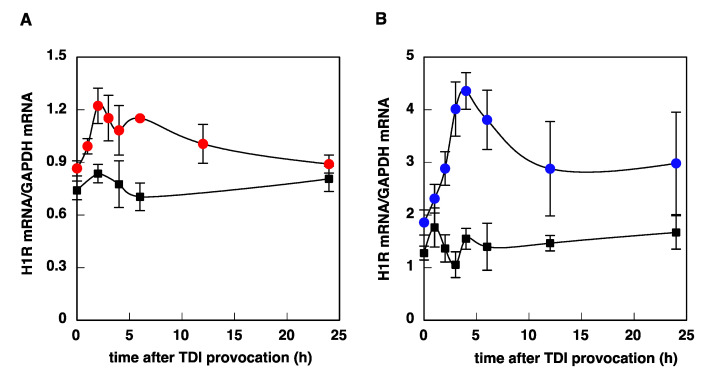
Time course of TDI-induced H1R mRNA upregulation in trigeminal ganglion (**A**) and nasal mucosa (**B**) of TDI-sensitized rats. At given time intervals after provocation with TDI, total RNA was isolated from trigeminal ganglion and nasal mucosa of TDI-sensitized and control rats. H1R mRNA was determined by real-time quantitative RT-PCR. Closed circles, TDI-sensitized rats; closed squares, control rats. Data are expressed as means ± SEM (*n* = 4).

We investigated the time course of histamine-induced upregulation of H1R mRNA in U-373 MG cells and HeLa cells. Stimulation with histamine caused a rapid and transient increase in H1R mRNA levels in U-373 MG cells, with a maximum at 2–3 h after stimulation (Figure 3A), whereas histamine-induced upregulation of H1R gene expression in HeLa cells was slow and gradual, and the expression level reached a maximum at 3–9 h (Figure 3B). The kinetics observed in U-373 MG cells and HeLa cells were similar to those in the trigeminal ganglion and nasal mucosa, respectively (Figure 4).

### 3.3. Transcriptional Regulation of H1R Gene Expression in U-373 MG Cells

Time course studies of histamine-induced H1R gene upregulation in HeLa cells and U-373 MG cells suggest that the transcriptional regulation of the H1R gene expression signaling pathway in neuronal cells is different from that in non-neuronal cells. Swan et al. demonstrated that three common 5’ terminal exon splice variants of human H1R, A/K, B/K, and F/K by 5’ rapid amplification of cDNA ends (RACE) [28]. We previously showed that the 2.1 kb DNA fragment from the upstream regulatory region of the human H1R gene expressed histamine- or PMA-induced promoter activity in HeLa cells [15].

**Figure 5:**
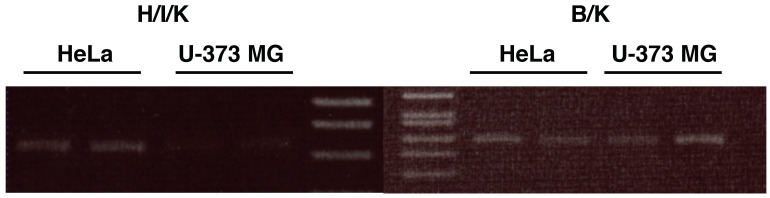
Expression of H/I/K and B/K splice variants in U-373 MG cells and HeLa cells. After starvation in serum-free medium for 24 h, total RNA was isolated. Total RNA (2 μg) was reverse-transcribed to cDNA in the reaction mixture of 20 μL. Three μL of aliquot was used for the endpoint-PCR analysis by using splice variant-specific primers shown in Materials and Methods.

The promotor assay revealed that two regions, designated region A (from −1137 to −960; +1 indicates the putative transcription initiation site [29]) and region B (from −65 to −44) are responsible for the promoter activity of the H1R gene [30], which corresponds to the putative promoter region for H/I/K splice variant in Swan’s paper. According to Swan’s paper, the B/K splice variant is abundant in brain-derived tissue [28]. In our preliminary studies, we showed that the expression levels of A/K and F/K variants are low in both cells (data not shown). Thus, in this study, we focused on the B/K splice variant. RT-PCR analysis confirmed that both B/K and H/I/K splice variants are expressed in HeLa cells, while B/K splice variant was detected in U-373 MG cells, but the expression of H/I/K splice variant was very faint in our PCR condition, indicating that the expression of this variant is very low, if any, in U-373 MG cells (Figure 5).

Next, we investigated whether the B/K splice variant is involved in histamine-induced upregulation of H1R mRNA in U-373 MG cells. In general, promoter activity is measured as luciferase enzyme activity. However, the level of the luciferase activity did not directly reflect the transcriptional level of the reporter gene, and the rapid change in transcriptional activity could not be analyzed by using the conventional reporter assay [31]. Thus, we investigated the kinetics of luciferase mRNA expression in response to histamine/PMA stimulation in both HeLa cells and U-373 MG cells.

**Figure 6:**
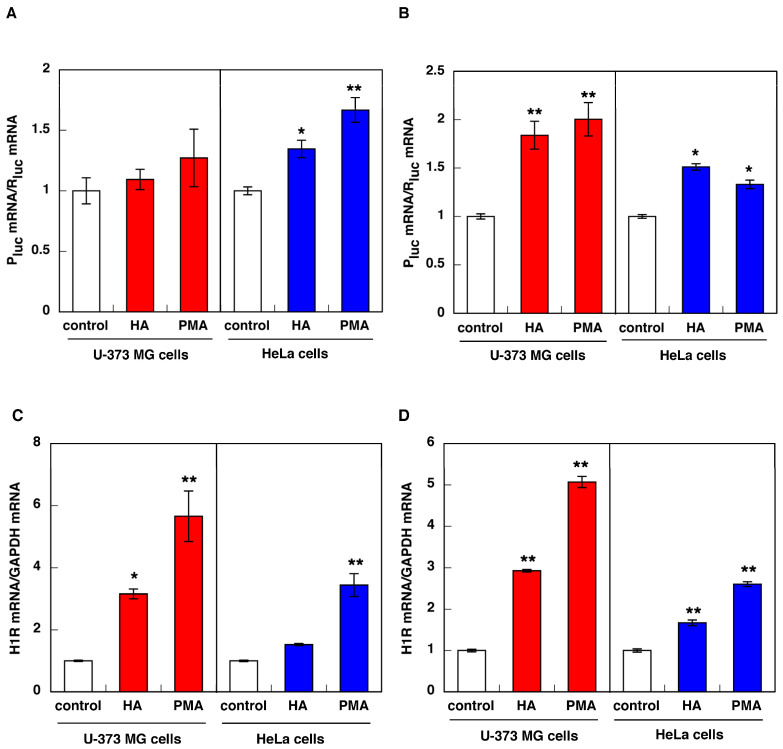
Activation of the promoter of H/I/K splice variant (**A**,**C**) or B/K splice variant (**B**,**D**) by histamine and/or PMA stimulation in U-373 MG cells and HeLa cells. Cells were transfected with the human H1R reporter plasmid containing the putative promoter region of H/I/K splice variant (**A**,**C**) or B/K splice variant (**B**,**D**) for 12 h. Then, the cells were starved in serum-free medium for 24 h before treatment with 100 μM (U-373 MG cells) or 10 μM (HeLa cells) histamine (HA) or 100 nM PMA. Luciferase mRNA (**A**,**B**) and H1R mRNA (**C**,**D**) was determined by quantitative RT-PCR 2 h (U-373 MG cells) or 3 h (HeLa cells) after histamine/PMA treatment. Data are expressed as means ± SEM (*n* = 6). ** *p* < 0.01, * *p* < 0.05 vs. control. For statistical analysis, one-way ANOVA with Tukey–Kramer’s test was used.

In HeLa cells, stimulation with histamine/PMA caused an increase in luciferase mRNA by the promoter assay using the luciferase vector containing 2.5 kb of 5′-upstream of exon H (Figure 6A right panel). However, stimulation with histamine/PMA failed to increase luciferase mRNA in U-373 MG cells (Figure 6A left panel). In both cells, H1R mRNA was upregulated by stimulation with histamine/PMA (Figure 6C). These data suggest that this promoter (corresponding to the putative promoter region for H/I/K splice variant) is responsible for histamine/PMA-induced upregulation of H1R gene expression in HeLa cells but not in U-373 MG cells. 

**Figure 7:**
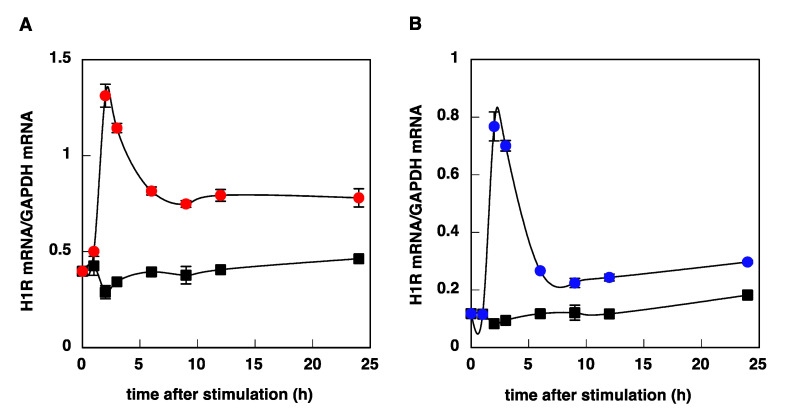
Time course of histamine-induced upregulation of H1R mRNA derived from B/K splice variant in U-373 MG cells (**A**) and HeLa cells (**B**). At given time intervals after stimulation with 100 μM (U-373 MG cells) or 10 μM (HeLa cells) histamine, total RNA was isolated, and B/K splice variant mRNA was determined by real-time quantitative RT-PCR using B/K specific primer and probe shown in Materials and Methods. Closed circles, B/K splice variant; closed squares, control. Data are expressed as means ± SEM (*n* = 6–9).

Next, in order to assess the possibility that the B/K splice variant is responsible for the histamine-induced rapid and transient increase in H1R mRNA expression in U-373 MG cells, we cloned the 2.5 kb DNA fragment upstream of exon B [28] and investigated the promoter activity of this putative promoter region. Treatment with histamine/PMA increased in the expression of luciferase mRNA (Figure 6B) and H1R mRNA (Figure 6D) in both cells, suggesting that this promoter region contains transcription factor binding sites responding to histamine/PMA stimulation. In addition, real-time quantitative RT-PCR analysis using B/K specific primer/probe showed that the kinetics of B/K splice variant are very similar to that of “total” H1R mRNA in both cells (Figure 7, compared to Figure 3). These data suggest that the B/K splice variant might be the transcript expressed in U-373 MG cells and is responsible for the histamine/PMA-induced rapid and transient increase in H1R mRNA expression in U-373 MG cells.

Recent human genomic sequencing research revealed that many genes contain multiple first exons, including protocadherin, UDP glucuronosyltransferase, plectin, neuronal nitric oxide synthase (NOS1), and glucocorticoid receptor (GR) genes [32]. Turner et al. also reported tissue-specific differential usage of the first exons in the human GR gene and suggested that alternative first exons, each under the control of specific transcription factors, control tissue-specific GR expression and are involved in tissue-specific GR transcriptional response to stimulation [33]. The BRCA1 gene was also reported to have two different exons and possesses transcriptional control via these alternative first exons involved in the downregulation of translation [34]. Similar translation regulation by different 5′-UTR usage was found in NOS1 mRNA [35]. These reports suggest that a repertoire of distinct usage of exon1 could influence post-transcriptional gene regulation, such as processing, export, stability, and translation of mRNA, and it also could regulate gene expression in response to a wide variety of signals. However, the effect of usage of alternative exon1 on the kinetics of the expression of the transcript variants has not yet been investigated. Thus, to our knowledge, this is the first report to show different uses of exon1 influencing the kinetics of the expression of the transcript variants. The mechanism underlying tissue-specific altered kinetics of expression for these variants require further study. Recently, we have reported that the A/K promoter might be responsible for IL-4 induced upregulation of H1R gene expression in HeLa cells, and this signaling is mediated by the JAK3-STAT6 pathway [22]. Moreover, we have reported that multiple transcription factors including AP-1 and ETS-1 are involved in the transcriptional regulation in HeLa cells, and this signaling is mediated by the PKCδ-ERK pathway [30]. The involvement of IKK/IκB/NF-κB signal pathway was also reported in histamine-induced proinflammatory cytokine production in human epidermal keratinocytes [36]. These findings indicate the complexity of the transcriptional regulation of H1R gene expression. In order to overcome this issue, the transcription factors specifically involved in the transcriptional regulation of each variant should be identified.

In conclusion, in the present study, we have shown that the promoter of the B/K splice variant [28] might be involved in a rapid and transient increase in H1R mRNA observed in U-373 MG cells, and the PKCα signaling pathway is mainly involved in this upregulation. On the other hand, the promoter of the H/I/K splice variant [28] might be involved in the slow and gradual upregulation of H1R gene expression found in HeLa cells, and the PKCδ signaling pathway is involved in this upregulation [15,16,30]. We have shown that activation of the gene expression signaling pathways for H1R gene followed by the elevation of their mRNA levels are responsible for the pathogenesis of allergic rhinitis [9,10]. However, changes in mRNA levels are often not reflected in protein levels. We have reported that histamine or PMA stimulation increased H1R at both mRNA and protein levels in HeLa cells [15]. In addition, it was reported that the strength of H1R signaling depends on the H1R expression level [37]. Thus, it is important to regulate H1R gene upregulation in order to alleviate nasal symptoms. These data also suggest that compounds that target PKCδ could work as peripheral-type H1R selective inhibitors without a sedative effect.

## Figures and Tables

**Table 1:** Oligonucleotide primers and probes used for real-time quantitative RT-PCR.

Sequence
H1R mRNA Forward primer: 5’-CAGAGGATCAGATGTTAGGTGATAGC-3’ Reverse primer: 5’-AGCGGAGCCTCTTCCAAGTAA-3’ Probe: 5’-FAM-CTTCTCTCGAACGGACTCAGATACCACC-TAMRA-3’
A/K variant mRNA Forward primer: 5’- AGCCCTGAGGTCTGGAGACAG -3’ Reverse primer: 5’- TTGCCCTCACAACATCTTGTCTTC -3’ Probe: 5’- FAM-TGAAACCAGCCAGGGAGTGAGCCATAC -TAMRA-3’
B/K variant mRNA Forward primer: 5’-CAAACTTTCCCCGGAGCCG-3’ Reverse primer: 5’-TGTTGCCCTCACACATCTTTGTCTTC-3’ ^1^ Probe: 5’-FAM-CTCCTGCCT-3’
F/K variant mRNA Forward primer: 5’-TTGCCAGGGTAAGAGGATGAG-3’ Reverse primer: 5’-AGGAATTGGGGAGGCTCATTG-3’ Probe: 5’-FAM-CAGGAGAGCAGCATTTGTAAAGGGAG-TAMRA-3’
*Photinus pyralis* luciferase (P_luc_) mRNA Forward primer: 5’-TGAGTACTTCGAAATGTCCGTTC-3’’ Reverse primer: 5’-GTATTCAGCCCATATCGTTTCAT-3’ ^2^ Probe: 5’-FAM-GGCAGAAG-3’
*Renilla reniformis* luciferase (R_luc_) mRNA Forward primer: 5’-GGAGAATAACTTCTTCGTGGAAAC-3’ Reverse primer: 5’-GCTGCAAATTCTTCTGGTTCTAA-3’ ^3^ Probe: 5’-FAM-TGTTGCCA-3’

^1^ Roche Universal Probelibrary probes #51. ^2^ Roche Universal Probelibrary probes #29. ^3^ Roche Universal Probelibrary probes #145.

**Table 2:** Oligonucleotide primers used for cloning of promoter.

	Sequence
A/K variant	
Forward primer	5’-CAAGACTGCTAGCTTAGCCAAGCAAGTTGG-3’
Reverse primer	5’-AGCAGTTAAGCTTCCAATCAGCCACCTCAGTC-3’
B/K variant	
Forward primer	5’- ACTAGTCTGCTTACCAGGGGCTTGAAATCATG -3’
Reverse primer	5’- AAGCTTAGGTGTCTGCGCGTCGAGTGCTG -3’
F/K variant	
Forward primer	5’-TGGTGGCTCGAGAATCCTTGCCCTGAAGACTG-3’
Reverse primer	5’-GTTGTTATAAGCAAACAGGTCTACTCC-3’

## Data Availability

Not applicable.
